# The effect of Functional Electrical Stimulation-assisted posture-shifting in bone mineral density: case series-pilot study

**DOI:** 10.1038/s41394-022-00523-9

**Published:** 2022-06-10

**Authors:** Monica Armengol, Ioannis D. Zoulias, Robin S. Gibbons, Ian McCarthy, Brian J. Andrews, William S. Harwin, William Holderbaum

**Affiliations:** 1 Centre of Biomedical Engineering, School of Biological Science, University of Reading, Reading, UK; 2 Centre for Rehabilitation Engineering and Assistive Technology, University College London, London, UK; 3 Pedestrian Accessibility and Movement Environment Laboratory, Department of Civil, Environmental and Geomatic Engineering, Faculty of Engineering Science, University College London, London, UK; 4 School of Engineering, University of Warwick, Coventry, UK; 5 Nuffield Department of Surgical Sciences, University of Oxford, Oxford, UK; 6 Department of Engineering, Manchester Metropolitan University, Manchester, UK

**Keywords:** Bone, Bone

## Abstract

**Study design:**

A training intervention study using standing dynamic load-shifting Functional Electrical Stimulation (FES) in a group of individuals with complete spinal cord injury (SCI) T2 to T10.

**Objectives:**

Investigate the effect of FES-assisted dynamic load-shifting exercises on bone mineral density (BMD).

**Setting:**

University Lab within the Biomedical Engineering

**Methods:**

Twelve participants with ASIA A SCI were recruited for this study. Three participants completed side-to-side load-shifting FES-assisted exercises for 29 ± 5 weeks, 2× per week for 1 h, and FES knee extension exercises on alternate days 3× per week for 1 h. Volumetric Bone Mineral density (*v*BMD) at the distal femur and tibia were assessed using peripheral quantitative computed tomography (pQCT) before and after the intervention study.

**Results:**

Participants with acute and subacute SCI showed an absolute increase of f trabecular *v*BMD (*v*BMD_TRAB_) in the proximal (mean of 26.9%) and distal tibia (mean of 22.35%). Loss of *v*BMD_TRAB_ in the distal femur was observed.

**Conclusion:**

Improvements in *v*BMD_TRAB_ in the distal tibia were found in acute and subacute SCI participants, and in the proximal tibia of acute participants, when subjected to anti-gravity FES-assisted load-bearing exercises for 29 ± 5 weeks. No *v*BMD improvement in distal femur or tibial shaft were observed in any of the participants as was expected. However, improvements of *v*BMD in the proximal and distal tibia were observed in two participants. This study provides evidence of an improvement of *v*BMD_TRAB_, when combining high-intensity exercises with lower intensity exercises 5× per week for 1 h.

## Introduction

After Spinal Cord Injury (SCI), secondary complications, such as rapid decrease in sub-lesional bone mineral density (BMD) concurrent with severe muscle atrophy occurs. Up to 40% of BMD can be lost within two years post-injury [1]. This reduction in BMD mainly occurs in the tibia [2], making individuals with SCI 5-23 times more likely to have a tibial or femoral fracture compared to the general population [3]. Additionally, fractures in individuals with SCI can be complex and have severe consequences, including increased morbidity [1], and high economic [4] and social cost [5]. Therefore, the design and implementation of rehabilitation therapies to improve or reduce bone loss in individuals with SCI are timely.

The mechanotranductor nature of the bone as a response to loading and muscle contractions, allows us to hypothesise, that under the right conditions bone can regenerate [6]. The effect of exercise in BMD has been investigated by many authors with mixed results. Functional Electrical Stimulation (FES) is a technique that uses low-level electrical currents to activate neural filaments, which if applied to the nerve endings associated with muscles, it causes the muscle to contract and therefore elicit movement.

FES training interventions sessions of 15–30 min, 3–5 days/week over 6 months, have shown no changes in BMD during FES cycling [7], or ambulatory walking for 12–20 weeks [8]. However, improvements in the distal femur and proximal tibia of 11% and 12% respectively with FES cycling [9], and BMD improvement of 30% in acute and chronic [10] individuals with SCI performing resistive static exercises were shown in other studies.

Although it is not clear the differentiating factor between these studies, parameters such as the dosage (time of exercise per week), the FES stimulator parameters (i.e., frequency, pulse duration), the force achieved while standing, and the type of exercise (resistive, passive, static or dynamic), could influence the effect over BMD.

The current study aims to investigate the effect of FES-assisted dynamic load-shifting exercise on BMD in the tibia and femur of individuals with SCI. Specifically the magnitude and pattern of joint contact forces (JCF) while loading.

## Methods

### Participants

Twelve participants with complete SCI (ASIA A) T2–T10 joined this study. Informed written consent to take part was given by all participants. The study was ethically reviewed and obtained approval from the National Health System (NHS) and the University of Reading (UoR) Research Ethics Committee. Inclusion criteria included adults age 18–65 years with complete motor and sensory ASIA A SCI between T1 and T10. Exclusion criteria included upper body motor disfunction that prevented full control of upper limbs, previous fractures of femur or tibia post SCI, severe autonomic dysreflexia, intake of medications that would prevent or enhance bone formation, and pregnancy.

### Equipment description

**Fig. 1:**
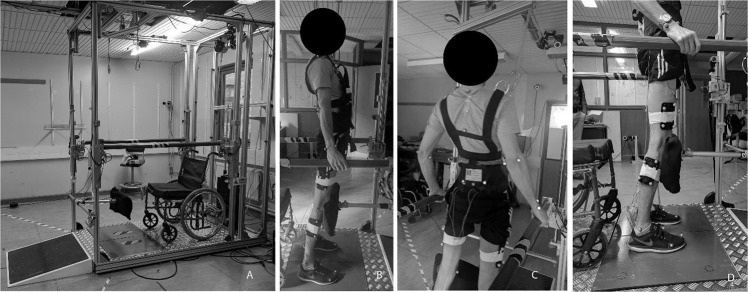
FES assistive standing Frame. **A** Novel standing system for load shifting exercises, **B**–**D** Participant standing for FES-assisted weight shifted exercises and infrared markers.

A smart standing exercise system [11] was developed in-house to allow participants to carry-out dynamic posture shifting securely (Fig. 1.A). The smart system consisted of a standing frame, a 16-channel FES stimulator, an exer-game (exercise game) platform, and a motion capture (MOCAP) system (Qualysis, Sweden). The smart frame had two force plates (4060, Bertec Corporation, Ohio, USA), two handlebars on each side of the frame which were mounted on six load sensors (S Type Load Cell (0–100 kg)–CZL301C, Phidgets Inc, Canada), which enabled measurement of the forces applied through the hands in three orthogonal directions. The frame also had a winch attached to a harness (Maine Anti-Gravity Systems Inc, USA) worn by the frame user. The 16-channel stimulator was developed in-house, and channels were controlled through a PC. The stimulator signal power was adjusted manually, followed by delivering pre-programmed stimulation in alternating patterns to swift weight from side-to-side. Patterns were synchronised with exer-game to guide movements.

### Protocol for exercices: training and intervention

The study was divided into two phases: phase one FES leg conditioning *(FES-LC)*, and phase two FES intervention (*FES-IP*).

#### FES Leg conditioning (FES-LC)

**Fig. 2:**
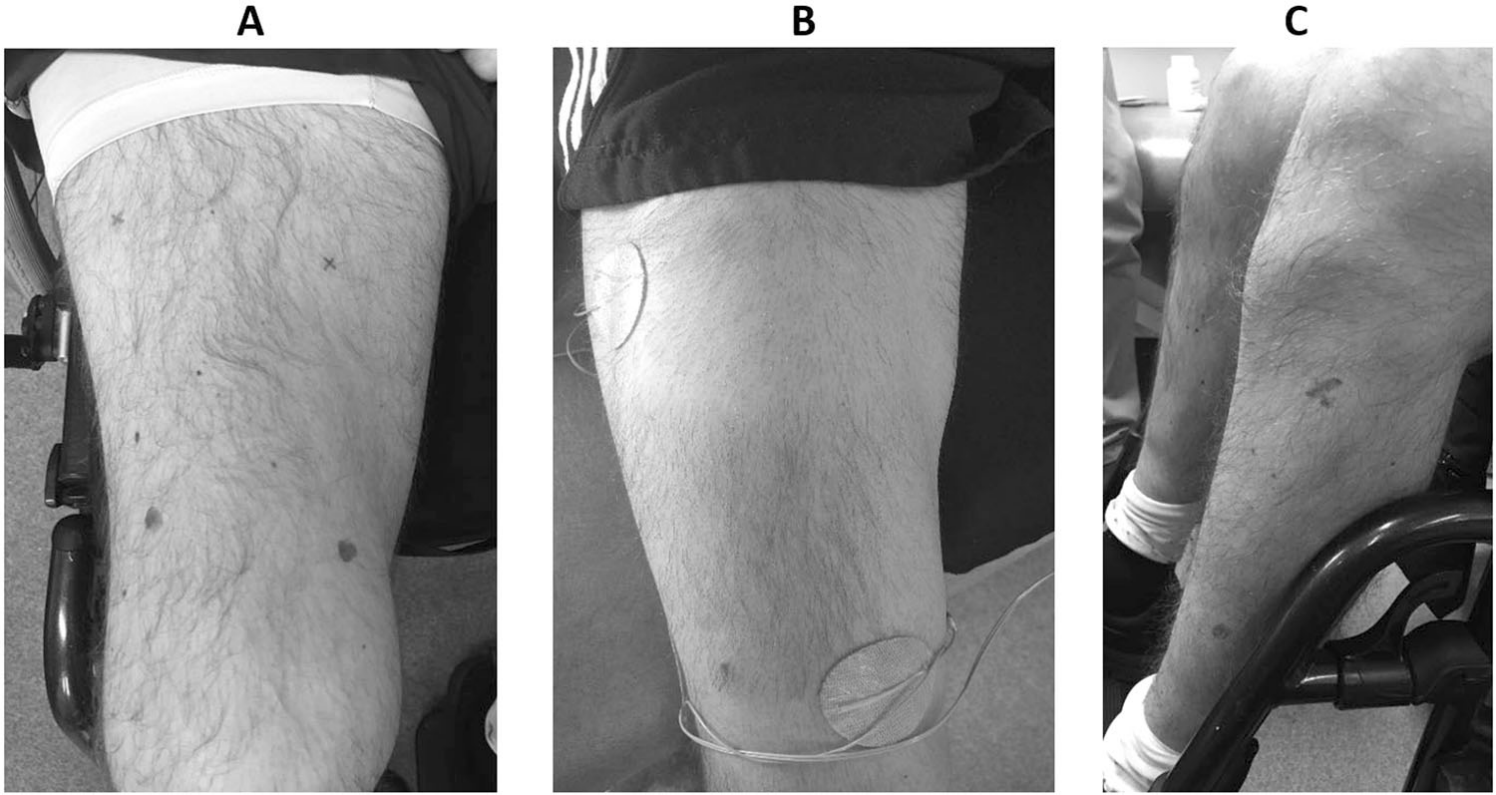
Motor Point identification in thigh and calf muscles. Mapping of motor points and positioning of electrodes. **A** Motor points over thigh muscles (rectus femoris, vastus medialis and vastus lateralis) that allowed extension of the leg. **B** Positioning of superficial electrodes for FES-IP phase. **C** Motor points over calf muscles (gastrocnemius and soleus) to allow shifting of weight to the front of the foot.

This phase aimed to condition and strengthen the targeted muscles used during the FES-IP using FES. These consisted on muscle strengthening exercises using FES stimulation and muscle conditioning through stretching exercises. FES-LC participants were trained to use a 4-channel FES stimulator (Odstock Medical Ltd, Salisbury, UK) and on the placing of superficial electrodes (PALS plus, Axelgaard Manufacturing Co, Denmark) over muscles in the thigh (rectus femoris, vastus medialis and vastus lateralis) and calf (gastrocnemius and soleus) at located motor points (Fig. 2). The Odstock stimulator was set to a pulse frequency of 50 Hz, a pulse width of 250–300 µs, an amplitude of 100–115 mA, and a duty cycle on the pulse train of 1:1 set at 6 s ON/OFF. The training consisted of isotonic contractions of selected muscles alternating between legs. Leg conditioning was home-based 60 min/day continuously, 5 days/week (Supplementary Materials 1). Leg extension duration and fatigue were assessed to monitor progress.

Stretching exercises aimed to elongate the hip flexors and hamstrings which generally shorten after SCI due to prolonged sitting and standing 2–3 days/week for a minimum of 15 min were encouraged.

Prior to starting the FES-IP phase, participants had to demonstrate the ability to carry out FES-LC continuously for one hour, while achieving full extension of their knee. Additionally, participants were encouraged to achieve passive standing balance for over 1 min on the laboratory standing frame using a padded knee support. Parallel bars and heel restraints were also used to help maintain the standing balance (Fig. 1C) in a C-shape posture (Fig. 1B). A loose safety harness was used to avoid collapse only and did not bear weight.

#### FES Intervention Phase (FES-IP)

##### Protocol

The same FES-LC motor point placements were used to position the surface electrodes for this phase. Specifically, quadriceps, gastrocnemius, gluteus, and tibialis anterior. The electrodes placed on the gastrocnemius muscle were used to enable weight shift to the front of the feet on the stimulated leg. An additional support to the core muscles was given either by using 2 channel FES stimulation (P1, P3) or by using a lumbar support belt (DonJoy, DJO Global, California, USA) (P2, P3). The wheelchair was backed into the smart-frame, electrodes leads were connected to the FES stimulator. Participants wore a safety harness to avoid collapse.

Prior to FES standing, participants were asked to sit to the front of the wheelchair cushion, with feet positioned on a pre-marked area on the force plates. When instructed, participants stood using their arms for lateral balance, while FES was simultaneously applied to the quadriceps. During this phase, the safety harness winch was turned ON as a safety mechanism. Once statically standing, participants were given time to readjust themselves to achieve balance. As soon as the participant indicated they were comfortable, the exer-game was started.

This phase (FES-IP) used an in-house 16-channel adjustable monophasic pulse FES stimulator. Pulse width set to 250-300 µs with a 4 s 1:1 ON/OFF duty cycle, alternating muscle stimulation from leg to leg.

Participants were instructed to shift their upper body weight (BW) from side-to-side following visual cues provided by the exer-game synchronously with FES stimulation. This side-to-side movement was incorporated into a training session, twice a week for 60 min at University facilities. On the other 3/4 days, home-based FES-LC training, standing, and stretching continued 60 min per day.

##### Data recorded during FES-IP

Ground reaction forces (grf) measured by the force plates, the timing and level of FES stimulation level, forces applied in the hand bars obtained from load cells and exer-game difficulty level were recorded. Additionally, every 3-4 weeks, 51 reflexive markers were placed on anatomical landmarks to record participant’s motion in the frame (Miqus M3, Qualisys AB) (Fig. 1C).

### Bone mineral density measurements

#### pQCT scans

**Fig. 3:**
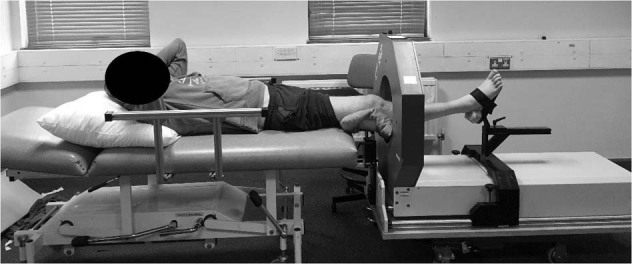
SCI participant lying supine in a pQCT scanner.

Bone measurements were carried out using a peripheral Quantitative Computed Tomography (pQCT) scanner (XCT 3000, Stratec Medical, Germany) (Fig. 3) which allows differentiation between the trabecular and cortical bone by collecting volumetric BMD (*v*BMD) data.

#### pQCT scan protocol

**Fig. 4:**
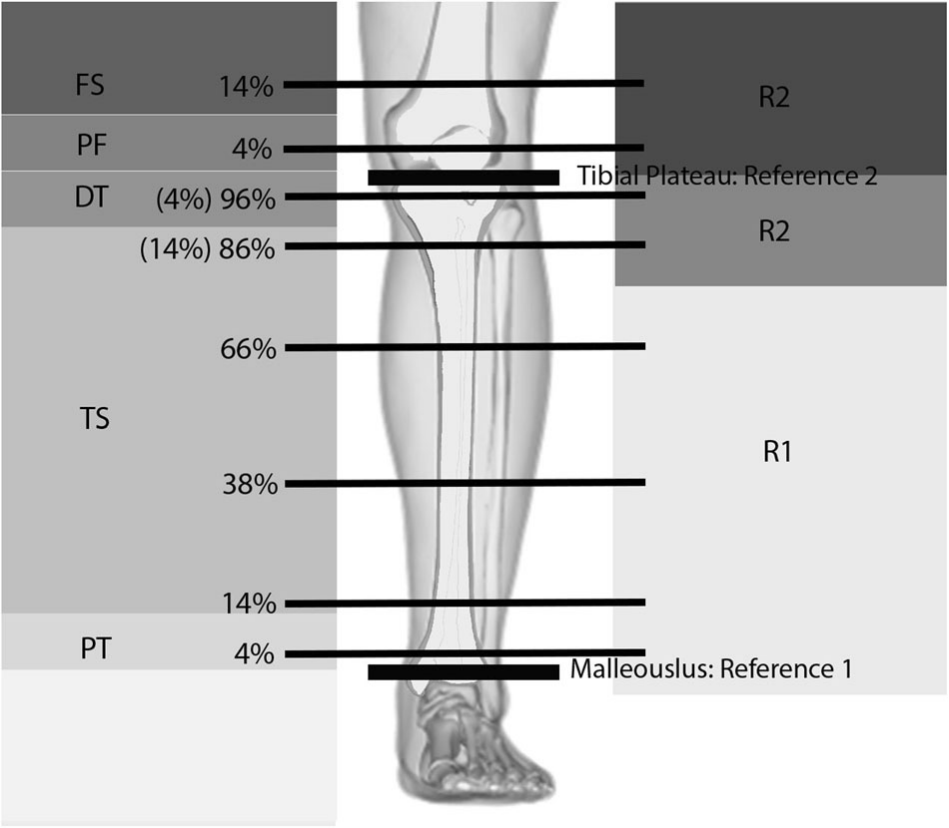
Scanned regions in the lower limb. Original photo of from CC BY-SA License [Internet]. 2020 was modified to include scanned sections.

Bone scans were acquired at two-time points; at the beginning and end of the FES-IP phase. Both legs were scanned, beginning with the most spastic leg reported by the participant. Scans at 4%, 14%, and 66% of the tibia (malleolus reference line) and 4% and 14% proximally and distally (femur) of the tibial plateau were carried out (Fig. 4). Each slice was 2 mm thick, with a speed of 30 mm/s, and images were taken with a voxel size of 0.4 for femoral scans and of 0.5 for all other scans to compensate for thinning of the cortical walls [2, 12].

### Biomechanical modelling of participants

Kinematic and force data recorded during the FES-IP, was divided into 20 s portions to allow for faster biomechanical analysis and used as input to the biomechanical model (OpenSim, Stanford, USA). The first and last 5 min of each session were disregarded.

A fully able-bodied model [13] was adapted to represent individuals with SCI, by de-activating all lower-limb muscles, except those stimulated by FES. Using static optimisation, JCF were calculated using the resultant forces and moments at each joint (Supplementary Materials 2. Appendix 1).

### Outcome measurements

#### Biomechanical model

The peak JCF and the progression over time was calculated. Specifically, the proportion of time where peak JCF exceeded 1.2x BW and 1.5x BW thresholds was determined by adding the recorded JCF of each joint as a percentage of the total time of the session.

#### pQCT scans

Trabecular and cortical *v*BMD were calculated in the different sections:

##### Epiphysis

The total *v*BMD (*v*BMD_TOT_), trabecular *v*BMD (*v*BMD_TRAB_) and total bone cross-sectional area (CSA_TOT_) were calculated. Thresholds of 180 mg/cm^3^ for the tibia and 150 mg/cm^3^ for the femur [14] to identify the periosteal surface were used. The trabecular bone was defined as 45% of the inner area.

##### Diaphysis

The cortical *v*BMD (*v*BMD_CORT_), cortical cross-sectional area (CSA_CORT_), the CSA_TOT_, and the bone mineral content (BMC) were calculated using a threshold of 710 mg/cm^3^ [2].

#### Processing BMD data

Mean values of BMD area was taken from pQCT scans at two-time points (t1, t2) at locations defined by patient-specific length of the femoral and tibial bone. Data from the right and left leg were averaged, except in cases where the scans from one leg were unusable (i.e., presence of metallic pins or plates, high movement artefact due to high spasticity, misplacement).

Bone loss functions developed by Eser et al. [12] where used to predict bone loss in individuals with SCI in the distal tibia (DT) and distal femur (DF) and the method was used to predict *v*BMD loss in the proximal tibia (PT).

## Results

### Participant recruitment and retention in the study

Twelve participants were recruited for this study (Table 1). However, only three participants completed both phases of the study (Table 2). The others withdrew from the study for different reasons (Supplementary Materials 1. Appendix 2).

**Table 1:** Participant demographics.

ID	ASIA	Injury level	Gender	Time post injury (1st visit) [yrs]	Age [yrs]
P1	A	T6	M	0.4	36
P2	A	T2	M	3.7	33
P3	A	T5	M	13.8	34
P4	A	T6	F	5.6	38
P5	A	T4	M	2.7	37
P6	A	T10	F	6.5	36
P7	A	T9	F	4.5	43
P8	A	T4	M	18.25	37
P9	A	T6	M	17.75	30
P10	A	T4	F	17.83	38
P11	A	T3	M	1.25	53
P12	A	T2	M	1.92	23

**Table 2:** Participant demographics who completed the study.

ID	ASIA	Injury level	Gender	Age (1st visit) [yrs]	Time post injury (at t1) [months]	Injury classification (@1st visit)
P1	A	T6	M	36	12	Acute (<2 yrs)
P2	A	T2	M	33	50	Subacute (2–4 yrs)
P3	A	T5	M	34	172	Chronic (>4 yrs)

Participants had complete SCI, ASIA A, with the level of injury between T2-T6. Average age was 34.7 ± 2 years old. Time post-injury was between 5–166 months at the time of the 1st visit.

### FES training compliance

FES-LC was completed in 17 ± 6 weeks, and FES-IP continued for a further 29 ± 5 weeks. Laboratory-based FES-IP compliance during P1, P2, P3 was 82%, 63%, and 78% respectively, and home-based FES-LC training for the three participants was 90%, 90%, and 30% respectively.

### Joint Contact Forces (JCF)

**Fig. 5:**
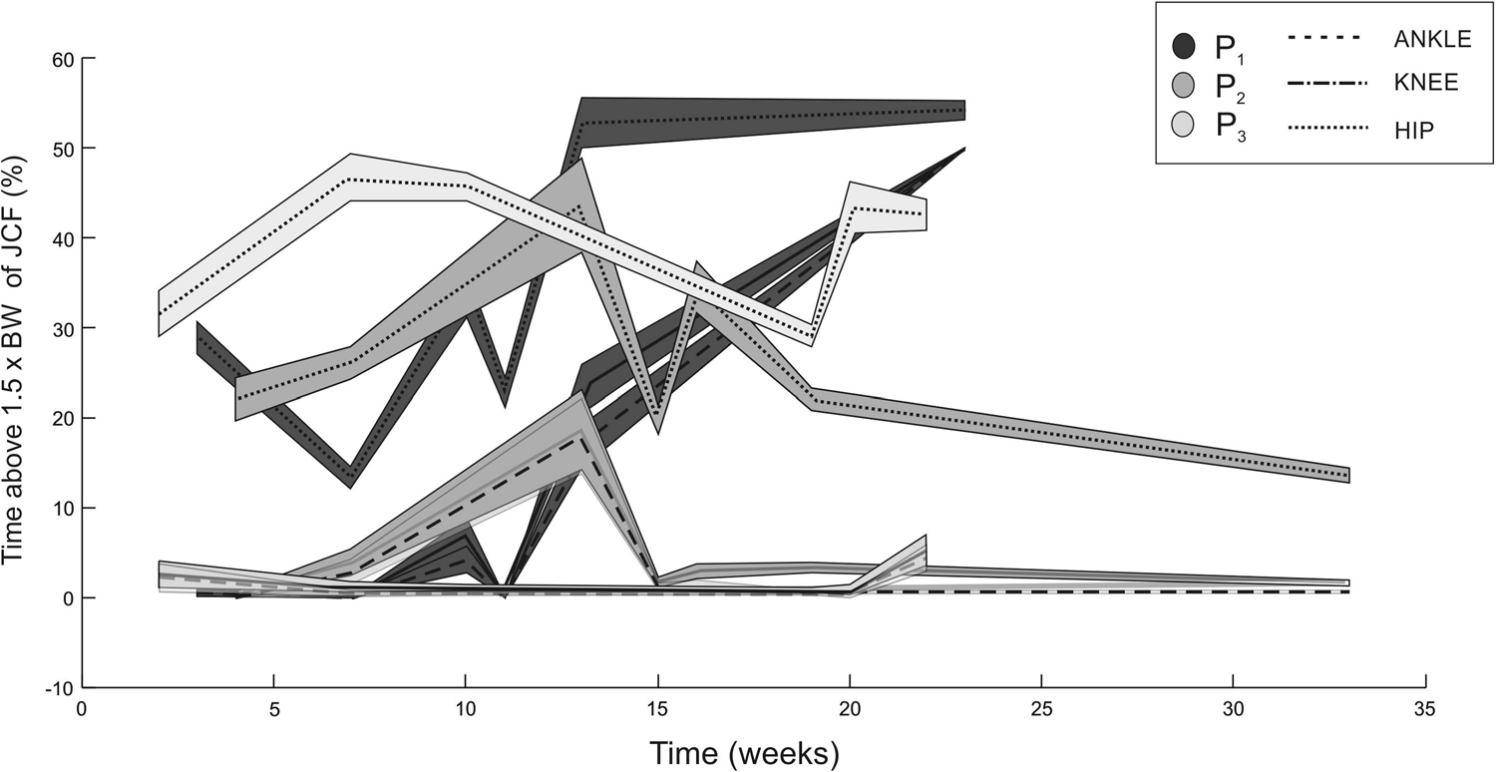
Portion of time (in percentage) that each participant spent above the 1.5 xBW threshold of JCF at each joint over time.

Participants P1 and P2 showed a significant increase in mean peak JCF (JCF_mp_) between weeks 10 and 15 in all joints. P1 showed the greatest overall improvement in JCF_mp_ and in the time above 1.5 xBW (Fig. 5), compared to the other participants. At the hip and ankle JCF_mp_ were significantly different (*p* = 0.0018). Participant P3 showed stable JCF throughout the sessions (*p* > 0.05).

Figure 5 shows the percentage of time where JCF was above 1.5× BW. At the hip JCF was above 1.5× BW for 20% of the time in all participants. Similarly, JCF at the knee and ankle were above 1.5× BW for 10% of the time for P1 and P2 between week 7 and 14, and less than 5% of the time for P3.

### Bone changes

#### Experimental vs. predicted vBMD values

Bone loss is described in Eq. 1, based on Eser et al. [12]. where *t* is time (years).

1 $$vBMD_{trab}\left( x \right) = \left\{ {\begin{array}{*{20}{l}} {DT:190.8\;e^{ - 0.4t} \;+ 65.2} \\ {DF:139.2\;e^{ - 0.56t} + 112.3} \\ {PT:110.8\;e^{ - 0.18t} + 105.3} \end{array}} \right.$$

**Fig. 6:**
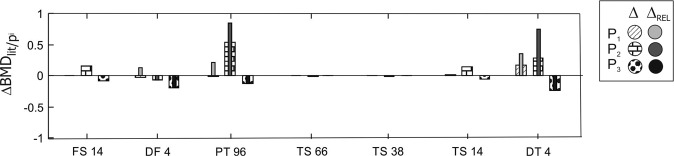
Changes in BMD (absolute and relative) of each participant in different sections of the bone: 14% of femoral shank (FS), 4% of the distal femur (DF), 4% of proximal tibia (PT) (or 96% of tibia), tibial shank (TS) at 66%, 38% and 14% from malleolus, and distal tibia (DT) at 4%.

Absolute changes in *v*BMD (Δ*v*BMD) obtained from experimental values for each participant ($\Delta {{{\mathrm{vBMD}}}}_{{{\mathrm{p}}}}$) and relative changes calculated from Eq. 1 [12] are shown in Fig. 6.

**Fig. 7:**
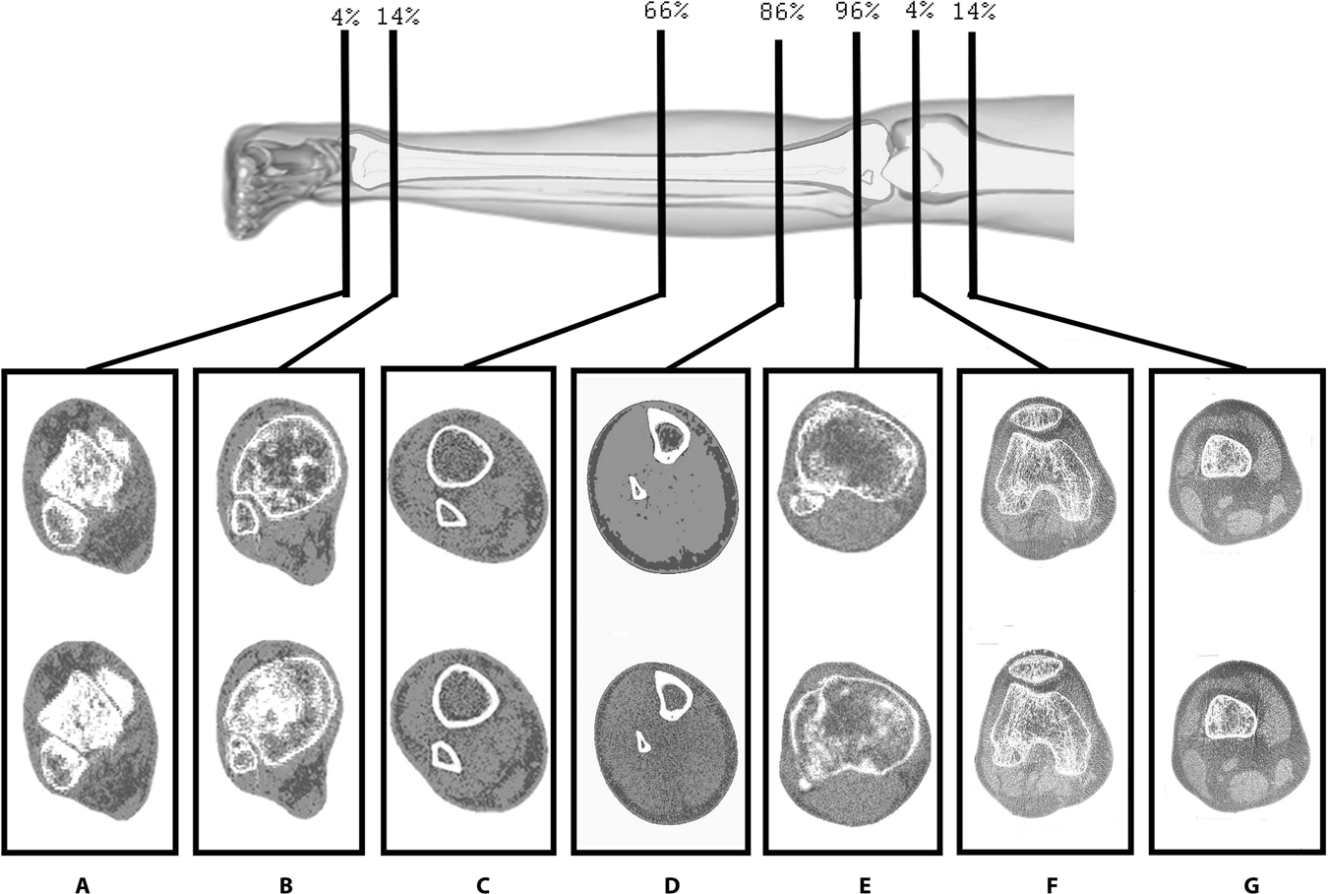
BMD changes at t1 (first row) to t2 (second row). Images correspond to pQCT sections of the Tibia 4%, 4%, 14%, 86% and 96% for **A**–**E** respectively and Femur 4% and 14% for **F**, **G** respectively. Reference lines for these scans were the malleolus for **A**–**D** and the tibial plateau for **D**–**G**. pQCT images **A**, **C**, **F**, **G** correspond to P1 and **B**, **D**, **E** correspond to P2.

Absolute *v*BMD loss in DF (*v*BMD_TRAB_) was observed in all participants (2.2%,7.4%,20.1%, for P1,P2,P3 respectively) (Fig. 7F). In relative terms, P1 showed 13% improvement of Δ*v*BMD_TRAB_. Additionally, there was an increase of Δ*v*BMD_TRAB_ at DT for P1 and P2 in absolute and relative terms (Figs. 6 and 7A, B). In the DT, P1 (16.8%) and P2 (27.9%) showed absolute improvements of Δ*v*BMD_TRAB_ while P2 showed loss *v*BMD_TRAB_ (8.9%). A small loss of Δ*v*BMD_TRAB_ was observed in PT for P1 (0.9%) and P3 (11,9%), while improvements of Δ*v*BMD_TRAB_ were apparent for P2 (52.9%) (Fig. 7E). Changes in cortical bone (Δ*v*BMD_CORT_) showed minimal change (<1%) in absolute values for all participants.

## Discussion

This study aimed to investigate the effect of FES-assisted dynamic anti-gravitational load-shifting exercises in BMD in the distal femur and tibia. This study provides some understanding of the effect of dynamic FES-assisted anti-gravitational load-bearing exercises and associated JCF forces on BMD in three individuals with complete SCI.

### Recruitment and retention of participants

In this study, 25% of participants completed the study. Similar studies in terms of commitment and time since injury have reported a small number of participants in total [15] or in each study group [16]. Other studies requiring “high-dose” training (>= 2 days/week) were carried out at home [17, 18] or participants lived close to the research facility. Home-based device for exercises has substantial advantages where exercise compliance is concerned.

### Biomechanics of standing and loading

Participant P3 was unable to achieve full knee extension while standing, although improvements in knee extension were observed. Therefore, an extra point of contact consisting of a sling supporting gluteus was used. This caused a backward shift of the centre of mass (CoM) and centre of pressure (CoP), which partially explains the reduced JCF achieved by this participant, by reducing the lever arm for an already decreased compressive force.

In this study, the JCF have been calculated from patient-specific motion and force data and processed using validated biomechanical tools modified to represent paraplegia.

### Bone mineral density

After approximately 29 weeks of exercise, it was possible to observe improvements in the epiphysis of the tibial bone, in both 4% DT and 4% PT. Improvements in *v*BMD_TRAB_ at PT have been observed in other studies that stimulated the quadriceps, during resistive knee extension, standing [16], or 50 rev/min upright cycling [18] in combination with other hamstrings and gluteus stimulation. These studies, with a variable length of 24 weeks to 3 yrs, showed BMD recoveries of up to ~30% in the trained leg versus the contralateral untrained control leg. Recovery of BMD at DT, an area also at risk of fracture in individuals with an SCI [2], was achieved by stimulating the soleus muscle 5× per week [17]. This study shows that by combining both stimulations, improvement in both DT and PT are achieved.

Recovery of BMD at DF has been observed in FES cycling exercise therapies [9, 19], by stimulating the hamstring muscles in combination to quadriceps and soleus/gastrocnemius muscle groups.

In the current study, no significant differences in the *v*BMD_CORT_ were found nor improvement in DF were observed. After an SCI, loss of trabecular bone occurs more rapidly than cortical bone [20] where initial structural changes are due to wall thinning [2].

Studies have shown that cortical bone can thicken if the bone is subjected to high strain levels [21] for prolonged times [22]. To the authors knowledge, no study with individuals with SCI and FES-assisted exercises has shown improvements in the cortical bone [23].

#### Thresholds for bone formation

Mechanotransduction properties of bone [24], suggest that there is are upper and lower threshold that favour bone restructuring [25]. In humans with SCI, compression loads of approximately 1.5× BW showed *v*BMD recovery, while the leg subjected to 0.4× BW showed no changes in *v*BMD [16].

In this study, we estimated JCF at the hip, knee, and ankle joints, using (a) real movement of each patient during the exercise sessions (MOCAP), and (b) a validated biomechanical model, modified to replicated stimulated muscles and surrounding muscles that allow movement to be possible. Using these tools, JCF and portion of time spent above different thresholds were calculated. In this study, participants P1 and P2 spent over 10% and 30% of the total time of sessions between weeks 7 to 14 above 1.5× BW, and 20% and 40% above 1.2× BW respectively in the same period. Nevertheless, these thresholds were not maintained for prolonged periods over FES-IP phase. Methodological tests with larger size groups should be performed to better understand the thresholds for bone formation and time they need to be sustained in BMD.

#### Frequency/dose of FES-stimulation or FES-assisted exercises

The frequency of FES stimulation application is also an important factor for bone restructuring. Studies that have shown BMD recovery or improvement were carried out for 30 to 60 min, 3× to 5× per week [17, 26]. Another study that showed 15% BMD recovery in the line of action of the muscle at 1.4× BW, used a dose of 10,000 pulse trains [26].

In this study, a 25% reduction in number of contractions per week was applied to participants compared to the Shields et al. study [17]. Participants in the current study were subjected to high compressive forces (>1.2× BW) during FES-IP twice a week for 1 h, and low forces (<1× BW) during FES-LC for another 3 days per week. Participants with high compliance in both FES-IP and FES-LC showed improvements in the DT and PT, while the participant with low training compliance did not show significant changes in *v*BMD.

These results suggest that the combined effect of low intensity and high-intensity exercises could create *v*BMD improvements. This finding can provide more flexibility in the exercise routines aimed at *v*BMD improvements in individuals with SCI, as FES-LC is easier to complete and requires less equipment.

### Secondary qualitative benefits of FES

Participants of this study reported improvements of thermic regulation while using FES and muscle hypertrophy. Additionally, participants reported physiological benefits such as better self-steam and empowerment, associated with standing and better stability while standing. FES is a technology with the potential to be portable and be used for any type of exercises that benefit the health of individuals.

### Future studies

This study suggests that combining low and high intensity exercises could be beneficial to bone regeneration. Further research with larger cohorts is needed to validate these findings.

## Supplementary information

Supplementary materials: The effect of Functional Electrical Stimulation-assisted posture-shifting in bone mineral density: Case series-Pilot study

Effect of stimulating thigh muscles with FES

## Data Availability

Data and materials are achieved following university and funding body guidelines and can be available on reasonable request.
