# Supplementary material for: The effect of Functional Electrical Stimulation-assisted posture-shifting in bone mineral density: case series-pilot study
Source: Spinal Cord Ser Cases. 2022 Jun 10;8:60. doi: 10.1038/s41394-022-00523-9 (PMC9184609; doi:10.1038/s41394-022-00523-9)
Supplement: Supplementary file 1 — Supplementary materials: The effect of Functional Electrical Stimulation-assisted posture-shifting in bone mineral density: Case series-Pilot study [file 41394_2022_523_MOESM1_ESM.docx]

Supplementary materials:

**The effect of Functional Electrical Stimulation-assisted posture-shifting in bone mineral density: Case series-Pilot study**

# **Appendix 1. Biomechanical Modelling**

A biomechanical model for SCI individuals was developed by adapting a fully able-bodied model [1]. The full able-bodied model consisted of 37 degrees of freedom and 80 Hill-type muscle-tendon units acting on the lower limbs. This model was used because although most of the stimulation occurs in the lower limbs, upper limb mass had to be considered to obtain passive balance (with no muscle contraction). In order to adapt the model to individuals with SCIs, the muscles in the original model were deactivated through an iterative process until a similar amount of muscles than those activated through FES in our study participants was obtained.

Motion data obtained at different stages of the intervention phase of the study, combined with the ground reaction forces (grf) and hand forces (hf), were used as input. Through static optimization analysis, the centre of mass of the body (bdCoM) and muscle activation was obtained. Results from static optimization results were assessed for residual forces and torques in the upper and lower body joints [2]. As the forces in the knee support were not measured, these forces were estimated by subtracting the bdCoM vector to the grf vector with the origin in the centre of pressure (CoP).

Joint contact forces (JCF) were calculated once an acceptable SCI model was obtained which (a) specified the number of muscles according to those being stimulated through FES in the intervention phase, (b) found non-trivial solution throughout the movement via the static optimization, and (c) maximised the input of muscles, rather than other help-forces (actuators).

# **Appendix 2. Information about participants that withdrew from study**

From the twelve participants recruited for this study, two did not respond to FES and therefore were not suitable to continue. One participant responded very little to FES, therefore direct stimulation through the femoral nerve was attempted for several months, however due to problems with keeping alertness through the day, the participant was not able to maintain regular training times and therefore muscles did not develop sufficiently to continue in the study. One participant withdrew from the study in early stages due to frequent signs of autonomic dysreflexia when using FES. Two participants withdrew due to time constraints to attend sessions and another three did not complete the training phase goals which made them unsuitable for the FES-IP. Leaving only three individuals who completed both the intervention and the training phase of the study.

**References**

1. Rajagopal A, Dembia C, DeMers M, Delp D, Hicks J, Delp S. Full body musculoskeletal model for muscle-driven simulation of human gait. IEEE Trans Biomed Eng [Internet]. 2016;63(10):2068–79.

2. Hicks JL, Uchida TK, Seth A, Rajagopal A, Delp SL. Is My Model Good Enough? Best Practices for Verification and Validation of Musculoskeletal Models and Simulations of Movement. J Biomech Eng [Internet]. 2015;137(2):020905.
