# Supplementary figures and images for: The effect of Functional Electrical Stimulation-assisted posture-shifting in bone mineral density: case series-pilot study
Source: Spinal Cord Ser Cases. 2022 Jun 10;8:60. doi: 10.1038/s41394-022-00523-9 (PMC9184609; doi:10.1038/s41394-022-00523-9)

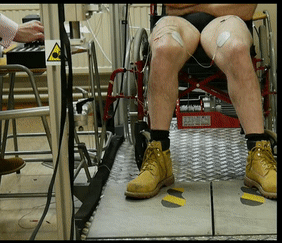

Supplement: Supplementary file 2 — Effect of stimulating thigh muscles with FES [file 41394_2022_523_MOESM2_ESM.gif]
